# Phthalates and diet: a review of the food monitoring and epidemiology data

**DOI:** 10.1186/1476-069X-13-43

**Published:** 2014-06-02

**Authors:** Samantha E Serrano, Joseph Braun, Leonardo Trasande, Russell Dills, Sheela Sathyanarayana

**Affiliations:** 1 Center for Child Health, Behavior and Development, Seattle Children's Research Institute, 2001 8th Avenue, 98121 Seattle, WA, USA; 2 Department of Epidemiology, Brown University School of Public Health, Providence, RI, USA; 3 Department of Pediatrics, New York University Langone Medical Center, New York City, NY, USA; 4 Department of Environmental Medicine, New York University Langone Medical Center, New York City, NY, USA; 5 Department of Population Health, New York University Langone Medical Center, New York City, NY, USA; 6 Department of Pediatrics, University of Washington School of Medicine, Seattle, WA, USA; 7 Department of Environmental and Occupational Health Sciences, University of Washington, Seattle, WA, USA

**Keywords:** Review, Phthalates, Diet, Food concentrations, Exposure, Daily intakes

## Abstract

Phthalates are associated with a variety of health outcomes, but sources that may be targeted for exposure reduction messaging remain elusive. Diet is considered a significant exposure pathway for these compounds. Therefore, we sought to identify primary foods associated with increased exposure through a review of the food monitoring survey and epidemiological data. A search in PubMed and Google Scholar for keywords "phthalates" and "diet" "food" "food stuffs" "dietary intake" "food intake" and "food concentration" resulted in 17 studies measuring phthalate concentrations in United States (US) and international foods, three epidemiological association studies, and three interventions. We report on food groups with high (≥300 μg/kg) and low (<50 μg/kg) concentrations and compare these to foods associated with phthalate body burden. Based on these data, we estimated daily intakes of di-2-ethylhexyl phthalate (DEHP) of US women of reproductive age, adolescents and infants for typical consumption patterns as well as healthy and poor diets. We consistently observed high DEHP concentrations in poultry, cooking oils and cream-based dairy products (≥300 μg/kg) across food monitoring studies. Diethyl phthalate (DEP) levels were found at low concentrations across all food groups. In line with these data, epidemiological studies showed positive associations between consumption of meats, discretionary fat and dairy products and DEHP. In contrast to food monitoring data, DEP was found to be associated with intake of vegetables in two studies. DEHP exposure estimates based on typical diets were 5.7, 8.1, and 42.1 μg/kg-day for women of reproductive age, adolescents and infants, respectively, with dairy as the largest contributor to exposure. Diets high in meat and dairy consumption resulted in two-fold increases in exposure. Estimates for infants based on a typical diet exceeded the Environmental Protection Agency’s reference dose of 20 μg/kg-day while diets high in dairy and meat consumed by adolescents also exceeded this threshold. The review of the literature demonstrated that DEHP in some meats, fats and dairy products is consistently found in high concentrations and can contribute to exposure. Guidance on future research in this area is provided that may help to identify methods to reduce dietary phthalate exposures.

## Background

Phthalates are a family of man-made chemicals that have applications in medical, automotive and consumer product industries [1]. Phthalates that are high-molecular weight, butylbenzyl phthalate (BBzP), di-2-ethylhexyl phthalate (DEHP) and mixtures of di-n-octyl phthalates (DnOP), are most well-known for their use as plasticizers in polyvinyl chloride (PVC) materials such as food packaging, flooring, and medical devices [2,3]. In recent years, di-nonyl phthalate (DiNP) and di-decyl phthalate (DiDP) have increasingly replaced DEHP in these applications [3]. Alternatively, low-molecular weight phthalates, dimethyl phthalate (DMP), diethyl phthalate (DEP) and dibutyl phthalate (DBP), are primarily added to cosmetics and personal care products as solvents, fixatives and adhesives [4]. Due to non-covalent bonds between the phthalate chemicals and their parent materials, there can be significant leaching and volatilization leading to environmental contamination and thus ubiquitous exposures in the general population. In fact, a recent report showed that metabolite biomarkers of eight major phthalates have been detected in 89% to 98% of the United States (US) population [3].

Phthalates are classified as endocrine-disrupting chemicals and have been linked to adverse health effects particularly in relation to early life exposures. Recent epidemiological studies have demonstrated significant associations between increased maternal urinary concentrations of metabolites of DEP, diisobutyl phthalate (DiBP), di-n-butyl phthalate (DnBP) and DEHP and shorter anogenital distance (AGD) in male infants, a marker of androgenization [5,6]. Prenatal exposures are also related to changes in timing of labor, infant hormone levels and infant and child neurobehavioral outcomes [4,6-12]. In adult populations, various epidemiological studies support an association between phthalate exposure and markers of testicular function in men, particularly decreased semen quality [13]. There is also evidence linking endometriosis in women with high phthalate metabolite levels [14]. Increases in waist circumference and body mass index (BMI) have been linked to DEHP, BzBP, DBP and DEP exposure in men and DEP exposure in adolescent and adult females [15,16]. One of the replacements for the DEHP, DiNP, has recently been designated as a carcinogen in the State of California [17].

Given the increasing scientific evidence base linking phthalate exposure with harmful health outcomes, it is important to understand major sources of exposure. A recent and well-designed study by Koch et al. [18] that monitored urinary phthalate excretion in individuals fasting for 48 hours, found that diet was the most significant pathway for exposures to DEHP, DiNP and DiDP while DMP, DEP, DiBP, DnBP and BBzP were primarily linked to non-food exposures [18]. According to a review by Cao, phthalates can migrate into food from plasticized PVC materials such as tubing typically used in the milking process, lid gaskets, food-packaging films, gloves used in the preparation of foods, and conveyor belts [19,20]. These compounds are also found in printing inks and adhesives on food wrappers as well as coatings on cookware that have been contaminated by packaging [20-22]. Foods high in fat are contaminated by higher weight phthalates that are more lipophilic such as DEHP [19]. In the United States, phthalates have been approved by the Food and Drug Administration (FDA) as plasticizers in food packaging materials and food contact substances used during processing and storage while the European Commission and Chinese authorities have limited phthalates in food contact materials made of plastic since 2008–2009 [21-23]. Thus, there can be substantial variability in phthalate concentrations within food groups based on the region of food production, processing practices, presence and type of packaging and lipid content [24,25]. With an ever increasing global market, phthalate contamination is a food safety issue that crosses international borders. Dietary phthalate exposure assessment has become a topic of great interest given the significance of the dietary pathway and health impacts associated with the specific phthalate species found in food. In recent years, an increasing number of food monitoring surveys and some epidemiology studies have addressed this issue; however a summary and analysis of these data together has not been conducted. Thus, we reviewed the food monitoring survey and epidemiology data on dietary phthalate exposure with the aim of identifying primary foods/diets associated with phthalate biomarker levels. We additionally calculated total daily intakes of dietary DEHP in the US population based on all available data from North America, Europe and Asia.

## Methods

We reviewed food monitoring studies and epidemiological papers on dietary phthalate exposure. In January of 2014, we searched in PubMed and Google Scholar for keywords "phthalates" and "diet" "food" "food stuffs" "dietary intake" "food intake" and "food concentration." The search resulted in three epidemiological studies, three interventions and 35 studies reporting phthalate concentrations in foods typically consumed by the general public. Papers in a language other than English were not reviewed. Further, food monitoring surveys were excluded if: 1) non-specific analytical techniques were utilized (e.g. flame ion detector (FID)/photo ion detector (PID) with packed columns) 2) if method validation was not reported (e.g. spiked samples) and 3) if no quality control measures were taken (e.g. process blanks). One publication combined the food phthalate concentrations of 14 studies [26]. The authors reported that "the problem of sample contamination during analysis generally [was] addressed" [26]. Therefore, studies that were included in these summary measurements were not individually reviewed. In total, 17 food monitoring surveys were included in this review.

We investigated phthalate species related to exposures through the diet as reported by Koch et al. 2013 (DEHP, DiNP, DiDP) and/or shown to be significantly associated with consumption of specific food groups in epidemiology studies (DMP, DEP, DnBP, DiBP, DnOP) [27,28]. Parent phthalate compounds and their primary metabolites are listed in Table 1.

**Table 1:** Phthalate parent compounds and their metabolites

**Phthalate name**	**Abbreviation**	**Urinary metabolite**	**Abbreviation**
Dimethyl phthalate	DMP	Mono-n-methyl phthalate	MnMP
Diethyl phthalate	DEP	Mono-ethyl phthalate	MEP
Di-isobutyl phthalate	DiBP	Mono-isobutyl phthalate	MiBP
Di-n-butyl phthalate	DnBP	Mono-n-butyl phthalate	MnBP
Di-n-octyl phthalate	DnOP	Mono-(3-carboxypropyl) phthalate	MCPP
Di-isononyl phthalate	DiNP	Mono-carboxyoctyl phthalate	MCOP
Di-isodecyl phthalate	DiDP	Mono-carboxynonyl phthalate	MCNP
Benzylbutyl phthalate	BzBP	Mono-benzyl phthalate	MBzP
Di-2-ethylhexyl phthalate	DEHP	Mono-2-ethylhexyl phthalate	MEHP
		Mono-(2-ethyl-5-hydroxyhexyl) phthalate	MEHHP
		Mono-(2-ethyl-5-oxohexyl) phthalate	MEOHP
		Mono-(2-ethyl-5-carboxypentyl) phthalate	MECPP

Within food monitoring studies, we first examined frequencies of detection as a percentage of positive food samples for each phthalate compound from total samples analyzed. We then compared frequencies across studies to indicate phthalate species with consistent high occurrence in food (i.e. at least half of studies reporting >50% detection of particular phthalates in all food samples) vs. low occurrence (<50% detection).

Most monitoring studies measured phthalate concentrations in similar foods which were then combined to report one summary measurement per food group/category (i.e. concentrations of white bread and wheat bread combined for one summary "bread" measurement). When not already presented in the publication, we calculated mean phthalate concentrations for food categories with non-detects equal to the limit of detection (LOD) divided by the square root of 2 or if available, the limit of quantification (LOQ) divided by the square root of 2. If specific values were not reported, then non-detects were set to zero. In our analysis of each food group, we only included measurements that were based off of more than one individual food sample (i.e. mean concentrations, composite sample measurements). In order to compare concentrations of food groups across studies, units were converted to μg/kg. Given the recent regulation set forth by the European Food Safety Authority (EFSA) to prevent phthalate contamination in food, phthalate food concentrations greater or equal to the specific migration limit (SML) for DBP of 300 μg/kg were considered high [23]. No such threshold exists under the FDA. Concentrations between zero and 50 μg/kg food were considered low since according to the EFSA, migration at this level reflects a low potential for exposure [29]. Concentrations greater than 50 and less than 300 μg/kg were designated as medium levels. To avoid overestimating phthalate levels in foods and potentially misclassifying foods as having high concentrations, we applied these criteria to average rather than maximum measurements of all phthalate species. We examined the phthalate species detected in particular food groups and classified foods as having low, medium, or high levels based on whether at least one of the toxicologically relevant phthalate species for reproductive effects (DEHP, DBP, BBzP, DiNP) was reported in greater than 50% of the summary measurements at the defined concentrations [30]. Prepared versus raw and canned versus fresh foods were reported separately. We then compared these results to those in epidemiologic investigations to see if similar findings were observed.

Since DEHP is prevalent in food and is among the most potent phthalate species for reproductive development, we calculated total dietary DEHP intakes on a daily basis (μg/kg-day) for US females of reproductive age (13–49 years) as a proxy for potential exposures to developing fetuses, adolescents (13–19 years) and infants (1–2 years) [31]. These groups may have the greatest susceptibility to the effects of DEHP given the sensitive windows of development in each life stage [6,32]. We examined dietary phthalate intake for each of these susceptible populations based on the consumption of eight main food groups (dairy, grains, vegetables, fruits, fats, meats, eggs, fish) each as a composition of total diet. We chose to examine four distinct dietary patterns to understand how increases and decreases in consumption of certain food groups impact dietary exposure. The four dietary patterns included 1) a diet reflecting average US consumption of these food groups based on 2003–2007 data of the National Health and Nutrition Examination Survey (NHANES) as reported in the US Environmental Protection Agency’s (EPA) Exposure Factors Handbook 2) a balanced diet based on recommendations by the USDA and the US Department of Health and Human Services in their document Dietary Guidelines for Americans 2010 (meats, eggs, fish combined to one protein category) 3) a diet with high consumption of fresh fruits and vegetables (excluding processed products) and 4) a diet with high consumption of meat and dairy. The latter two dietary patterns were based off of fruit/vegetable and meat/dairy consumption by individuals above the 90^th^ percentile within the United States Department of Agriculture’s (USDA) Continuing Survey of Food Intake by Individuals (CFSII) 1994–1995, 1998 as reported in the US EPA’s Exposure Factors Handbook [33,34]. Mean daily consumption rates were expressed in g/kg body weight per day. We calculated one weighted average DEHP concentration for each food group using all reported mean values (Additional file 1: Table S1). We used the following formula to perform exposure calculations.

$$\begin{array}{ll} \;\text{DI}\;\left(\mu g/\text{kg}-\text{day in food}\right) & =C\;\left(\mu g/\text{kg in food}\right)/1000*\\ & \;\text{CR}\;\left(g\;\text{of food}/\text{kg bw}-\text{day}\right) \end{array}$$

C is the phthalate concentration for each food group (μg/kg) and CR is the daily consumption of that particular food group (g/kg-day). DI is the daily intake of DEHP for each food group. Total intake was calculated as the sum of phthalate intake (μg/kg-day) for the eight food groups. An example is below for grain and fruit.

$$\begin{array}{ll} \;\text{Total DI}\;\left(\mu g/\text{kg}-\text{day}\right) & =\;\sum \;\text{DI}\;\left(\mu g/\text{kg}-\text{day in grain}\right)\\ & +\text{DI}\;\left(\mu g/\text{kg}-\text{day in fruit}\right). \end{array}$$

## Results

### Food monitoring studies

Seventeen food monitoring surveys published in North America, Asia and Europe between 1990 and 2013 were identified and reviewed [25,26,35-49]. Food phthalate concentrations by country are summarized in Additional file 1: Table S1.

In general, DiBP, DnBP, BBzP and DEHP were frequently detected in a variety of foods across food monitoring studies (>50% detection of food samples in at least half of studies) while DEP, DMP, DnOP and DiNP had low occurrence (0- 49% detection). In contrast to other countries, DMP and DEP were detected at high frequencies of 82% and 81%, respectively, in market food samples in China while a US study detected DEP in 57% of samples, second to DEHP (74%) [25,36]. Concentrations for DMP and DEP in these studies, however, were generally low in comparison to other phthalate species (<LOD – 25.98 μg/kg). Of the limited studies that investigated DiDP, only one fish sample was found to contain levels above detection limits in the United Kingdom (UK). Due to analytical methods of this particular UK study, high LOD and LOQ values ranging from 42.6 to 9489.5 μg/kg and 28.2 to 6243.9 μg/kg were reported for DiNP and DiDP, respectively, which resulted in high mean calculations of food concentrations [38].

#### Foods with consistent reports of high phthalate concentrations

**Meats: poultry** Investigators primarily analyzed beef, poultry and pork, individually, as well as these and other meats in combination. For poultry, all phthalate species but DiDP were detected. More than half of mean DEHP measurements were greater than 300 μg/kg while concentrations for other phthalates were generally low. In comparison to other meats, DEHP content in beef was variable, ranging from the limit of detection in United States samples to 1100 μg/kg in Canada [25,35]. Pork was found to have detectable levels of DEHP in all but one of the reviewed studies and some measurements approached the 300 μg/kg threshold. When meat products were analyzed in combination (beef, poultry, pork, other meats), most average DEHP concentrations approached high levels (175.8-758.3 μg/kg). All other phthalate species were reported at lower concentrations. Interestingly, a Canadian study reported high DnBP and DEHP concentrations in non-frozen packaged meat products while no phthalate species were detected in the frozen foods [35].

**Oils and fats** All phthalates were detected in oils and fats including butter, margarine, cooking oils and animal fats such as lard. More than half of mean DEHP concentrations were high across surveys ranging from 404 to 5,591.7 μg/kg. Levels varied for DnBP and BBzP with the greatest average concentrations at 3,287.5 and 11,083 μg/kg in Canadian samples [45]. All other phthalate species were found at lower levels for this food group.

**Dairy: cream** Although DEP, DMP, DnOP, DiNP and DiDP were not detected in any cream samples, high DEHP concentrations were observed from 413.1 to 1300 μg/kg. All other phthalates were reported at lower levels. In comparison to other dairy products, ice cream and cheese measurements approached (and sometimes exceeded) the threshold of 300 μg/kg. In cheese, all phthalates except for DnOP and DiDP were detected with DEHP levels ranging from 139.2 to 2270.6 μg/kg.

#### Foods with consistent reports of low phthalate concentrations

**Dairy products: yogurt, milk, eggs** Dairy products with low concentrations included yogurt, milk, and eggs. All phthalates except for DnOP, DiNP and DiDP were detected in eggs with low concentrations across studies. In yogurt products, only DEHP, BBzP and DMP were detected. Most BBzP and DMP measurements were reported at low mean concentrations from the limit of detection to 8.4 μg/kg and the limit of detection to 11.7 μg/kg, respectively. All phthalate species but DiNP and DiDP were detected in milk at low mean concentrations (<50 μg/kg).

**Grain: pasta, noodles and rice** Among grain products, pasta, noodles and rice were consistently contaminated with low levels of all phthalates that were detected including DiBP, DnBP, DEHP, DEP, BBzP, DMP and DnOP.

**Fruits and vegetables** Although all phthalate species except for DiNP and DiDP were detected in fruits and vegetables, concentrations across studies were generally low. In fresh vegetables, DEP concentrations ranged between the limit of detection and 9 μg/kg and between the limit of detection and 48.1 μg/kg in fruits. For DMP, two studies reported average levels of 10.3 and 13.5 μg/kg in fruits of China and France, respectively [36,40]. As a whole phthalate content was reported at minimal values for these food groups. Concentrations of all phthalate species in the few canned fruit and vegetable samples measured were comparable to fresh produce. However, vegetables and fruits found in jars were significantly higher than fresh produce with concentrations of 490.6 in pickles and 181.7 for jams and jellies [35].

**Beverages and water** As a whole, beverages and water had low phthalate levels. Highest average concentrations were observed for DnBP in Chinese beer (155.8 μg/kg) and for DEHP in Canadian juice (183 μg/kg) [35,36]. One study reported on both tap and bottled water and showed comparable phthalate levels with the highest for DEHP (12 μg/kg) [26].

#### Foods with reports of varied (Low, Medium, High) phthalate concentrations

**Seafood** All phthalate species were detected in seafood generally in low concentrations though DEHP was found at variable levels. Concentrations were detected from a minimum of 13 μg/kg in summary fish and seafood samples from Europe, North American and Asia up to levels as high as 928.6 μg/kg in frozen fish samples from Canada.

**Grain: bread and cereal products** All phthalates but DiNP and DiDP were found in grain products. Although concentrations varied within the cereal/cereal products subcategory, average values for DiBP, DnBP and DEHP were on the higher end while all other phthalate species were low. Many measurements of DEHP in bread products also approached and some exceeded the 300 μg/kg threshold.

**Spices** DEHP levels in spices varied among three studies with the highest concentration reported at 2,598 μg/kg. In this same study, DiBP, DnBP and BBzP concentrations also exceeded the 300 μg/kg migration limit, however, levels in the two other studies were low for these particular phthalate species [26,36,44].

### Epidemiological studies on dietary phthalate exposure

Three cross-sectional epidemiology studies were reviewed and summarized in Table 2[27,28,50]. Two studies assessed exposure through urinary phthalate metabolite measurements while one study calculated internal exposures by combining data on concentrations in various types of foods with exposure scenario assumptions. A cross-sectional study of 1,183 Swiss-German adults in Switzerland (mean age = 52.8 years) found that participants in the pre-defined "Fatty, Sweet and Ready Meal" cluster had higher exposures to DEHP and DBP than the other three clusters ("Healthy and Natural", "Health and Supplements", "Health Passive") (p < 0.01). The "Healthy and Natural" cluster was associated with higher BBzP exposures in comparison to other diets which the authors attributed to the consumption of bread. Finally, all clusters showed low exposure to DEP with participants in the "Health Passive" category having the lowest exposure in comparison to others (p < 0.01) [50].

**Table 2:** Epidemiological studies on dietary phthalate exposure

**Author/year (location)**	**Study design**	**Population/sample size**	**Outcome**	**Significant results**	
				**Positive associations/correlations**	**Negative associations/correlations**
Dickson-Spillman et al. [50] (Switzerland)	Cross-sectional	German-Swiss general population (n = 1183)	Relationship between diet clusters and calculated internal phthalate exposure	DEHP - "fatty, sweet, and ready meal" cluster > all others	DEP – "health passive" < all others
				DBP - "fatty, sweet, and ready meal" cluster > all others; "healthy and supplements">"health-passive"	
				BzBP - 'healthy and natural" cluster > all others	
Ji et al. [51] (Korea)	Intervention – Quasi experimental	Participants in Temple Stay program (n = 25)	Influence of strict vegetarian diet on urinary phthalate metabolite concentrations	5-oxo-MEHP - dairy	5-oxo-MEHP (females only) - vegetarian diet
				5-OH-MEHP - dairy	5-OH-MEHP - vegetarian diet
					MEP - vegetarian diet
					MnBP - vegetarian diet
					MiBP - vegetarian diet
Colacino et al. [27] (USA)	Cross-sectional	2003-2004 NHANES	Association between consumption of various types of foods and urinary phthalate metabolite concentrations	MEHP - eggs, poultry	MEHHP - fruit
		(n = 2374)			MEOHP - fruit
				MEHHP - poultry	MECPP - fruit
					Sum DEHP metabolites - fruit
				MEOHP - poultry	MBzP - fruit, tomatoes
					Sum of high molecular weight phthalates - fruit
				MECPP - poultry	
				Sum DEHP metabolites -poultry	
				MCPP - dairy	
				Sum of high molecular weight phthalates - poultry	
				MEP - potatoes, tomatoes, total vegetables, meat	
				MiBP - fish	
				MnMP - fruit	
				Sum of low molecular weight phthlates - tomatoes, total vegetables	
				Total phthalates - total vegetables	
Rudel et al. [52] (San Francisco Bay Area, CA, USA)	Intervention – Quasi experimental	Families w/two adults and two children	Contribution of food packaging to DEHP and BPA exposure through fresh, organic, and plastic-free dietary intervention		MEHP - intervention
		(n = 20)			MEHHP - intervention
					MEOHP – intervention
					(no changes in MEP, MBP, MBzP)
Sathyanarayana et al. [53] (Seattle, WA, USA)	Intervention –Two-arm, randomized study	Families w/two adults and two children	Efficacy of fresh, organic and plastic-free dietary intervention to reduce phthalate and BPA exposures	MEHP - intervention; dairy, spices	
				MEHHP - intervention; dairy, spices	
		Arm 1 – Intervention (n = 21)		MEOHP - intervention; dairy, spices	
				MECPP - intervention; dairy, spices	
		Arm 2 - Control (n = 19)		Sum DEHP metabolites – intervention; dairy, spices	
				MEP - intervention	
				MBP - intervention	
Trasande et al. [28] (USA)	Cross-sectional	2003-2008 NHANES children and adolescents (n = 2743)	Association between consumption of various types of foods and urinary phthalate metabolites in children and adolescents	MEHP - meat/poultry/fish, caloric intake; poultry	MEHP - fruit
					MEHHP - fruit, soy; soy
					MEOHP - fruit, soy; soy
					MECPP - soy
					Sum DEHP metabolites - fruit; soy
					MBzP - fruit
					MCPP - soy
					Sum of high molecular weight phthalates - fruit; non-citrus fruit, soy
					MEP - fruit, grain; fruit
					MiBP - caloric intake
					MBP - caloric intake
				MEHHP - meat/poultry/fish, caloric intake; poultry, discretionary fat	Sum of low molecular weight phthalates - fruit, grain; citrus fruit
				MEOHP - meat/poultry/fish, caloric intake; poultry, discretionary fat	
				MECPP - meat/poultry/fish, caloric intake; discretionary fat	
				Sum DEHP metabolite concentrations - meat/poultry/fish, caloric intake; caloric intake, poultry, discretionary solid fat	
				MCPP - meat/poultry/fish; discretionary fat	
				Sum of high molecular weight phthalates - meat/poultry/fish, caloric intake; caloric intake, poultry, discretionary solid fat						
				MEP - vegetables						
				MiBP - meat/poultry/fish						
				Sum of low molecular weight phthalates - vegetables						

Colacino and colleagues examined the contribution of different food types to phthalate exposure in 2,384 individuals (ages 6–85) within 2003–2004 NHANES [27]. The strongest associations between metabolite concentrations were observed with the intake of eggs and one DEHP metabolite (MEHP) (β: 0.145; 95% Confidence Interval (CI): 0.057, 0.232) and vegetables and MEP (β: 0.142; 95% CI: 0.072, 0.213). Assuming that the authors utilized a natural log transformation, this translates into a 15.6% increase in MEHP (95% CI: 5.87%, 26.11%) per unit consumption of eggs and 15.26% increase in MEP (95% CI: 7.47%, 23.74%) per unit consumption of vegetables. Increases of between 2.53% and 5.97% in metabolite concentrations were found per one additional ounce/cup of food for the following: poultry and the sum of DEHP metabolites and high-molecular weight phthalates; meat and MEP; fish and MiBP, dairy and MCPP; and fruit and MnMP. Inverse associations were found between sum DEHP and high-molecular weight metabolites and fruit.

A similar cross-sectional study conducted by Trasande and others assessed the relationship between food and caloric intake (assessed by 24-hour dietary recalls) and log-transformed urinary phthalate metabolites in 2,743 children and adolescents (ages 6–19) from NHANES 2003–2008 [28]. As a whole, analyses revealed that higher consumption of discretionary solid fat, meats and caloric intake was associated with increased high-molecular weight metabolite and sum DEHP metabolite levels; while increases in low-molecular weight metabolite levels were related to consumption of vegetables. We observed a 0.09% increase of high molecular weight phthalates, a 0.02% increase in DEHP metabolites, a 0.11% increase in MEHP, a 0.10% increase in MEHHP and a 0.10% increase in MEOHP per unit consumption of meat, poultry, and fish intake. In alternative models, each additional gram of poultry intake was associated with 0.19% increment in high molecular weight phthalates and 0.23% increase in DEHP metabolites. A non-significant increase of 0.37% in high molecular weight phthalates with each additional gram of organ meat intake was also reported. High and low molecular weight metabolite levels were negatively associated with fruit consumption.

Taken together, results from the three epidemiology studies provide evidence for associations between consumption of meats and fatty foods including dairy with DEHP metabolites and other high molecular weight metabolites like MCPP. Dickson-Spillman and others (2009) also reported similar associations with DnBP while two studies consistently showed a relationship between dietary intake of fish and increased concentrations of MiBP [50]. Consumption of vegetables was associated with increased DEP exposure in two studies by Colacino et al. and Trasande et al. while Dickson-Spillman et al. showed levels to be low across all diet clusters, including those with high consumption of fruits and vegetables [27,28,50]. Consumption of fruits appeared to be related to decreased DEHP metabolite levels.

### Intervention studies to reduce dietary phthalate exposure

Three interventions aimed to reduce dietary phthalate exposure were reviewed and summarized in Table 2. Ji and colleagues carried out a quasi experimental pilot intervention of a strict vegetarian diet on urinary phthalate metabolite concentrations in 25 participants in a five-day Buddhist Temple Stay program in Korea [51]. A 48-hr diet recall survey on participants’ dietary patterns prior to the temple stay was administered to understand routine intake of beef, pork, chicken, dairy, sheep, goat, duck, turkey and seafood (measured in servings/day). Dairy consumption was significantly and positively correlated with two secondary metabolites of DEHP, 5-oxo-MEHP (r = 0.33; p < 0.05) and 5-OH-MEHP (r = 0.31; p < 0.05). Further, levels of MEP, MnBP, MiBP, and 5-OH-MEHP were significantly decreased during the Temple Stay program in both men and women while a significant decrease in 5-oxo-MEHP was observed in females only. The authors concluded that dietary change, even in the short term could significantly reduce dietary exposure to phthalates [51].

An intervention study that aimed to evaluate the contribution of food packaging to phthalate exposure through a catered diet of fresh and mostly organic foods prepared without plastics was conducted in twenty people from the San Francisco Bay area [52]. Meals were prepared without any plastics in procurement, cooking, and serving.

The intervention was found to reduce geometric mean concentrations of MEHP by 53%, MEOHP by 55%, and MEHHP by 56% but no significant changes were observed in MEP, MBP or MBzP metabolites. At follow-up time points after the intervention, geometric mean concentrations of DEHP metabolites increased in participants, however the results were not statistically significant. The authors indicated that based on participant diaries of foods consumed before and after the intervention, potential exposure sources included meals prepared outside the home, canned foods, canned soda, frozen dinners, drinking from polycarbonate water bottles and microwaving in plastic. However, given that diaries were not comprehensive, other important sources may have been omitted [52].

In a similar intervention study, conducted by Sathyanarayana and others, 5 families (N = 20) from the Seattle area were randomized to receive fresh, organic foods prepared without plastics for five days (Arm 1) or educational hand-outs (Arm 2) instructing on how to reduce phthalate exposures [53]. An unexpected increase of median sum DEHP metabolite levels of 283.7 nmol/g at baseline to 7027.5 nmol/g was observed in the prepared foods group while no change was observed in educational handout group. Statistically significant increases were also observed for MEP, MBP and individual DEHP metabolites of MEHP, MEHHP, MEOHP and MECPP. DEHP metabolite concentrations decreased to baseline levels after the dietary intervention ended. Analysis of the catered foods used in the intervention showed very high levels of DEHP in dairy products as well as ground coriander that were suspected of contributing to the increase in metabolite levels.

### Estimation of dietary intake

**Figure 1:**
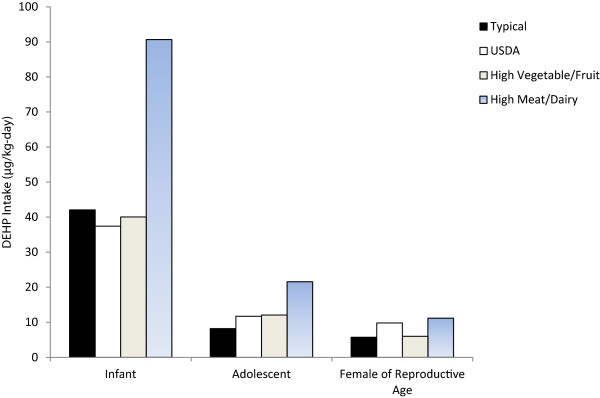
Per capita total DEHP intake (μg/kg-day) for four dietary patterns.

Among the eight food groups in actual dietary patterns, dairy intake was reported at the highest rate for all groups while fish and egg consumption was minimal (Table 3). Similarly, dairy is consumed at the greatest rate in USDA diets while oil and solid fat intake is minimized. Total dietary intake for DEHP based on actual dietary patterns was calculated at 5.7 μg/kg-day for women of reproductive age with the greatest contribution to exposure from dairy products (47.2%). For a 70 kg woman, this translates to a total exposure of 399 μg/day. The estimate for the daily intake of DEHP based on the recommended USDA diet was 9.8 μg/kg-day with dairy also contributing to the greatest exposure (68%). A diet high in meat and dairy resulted in the greatest DEHP exposure estimate, 11.2 μg/kg-day (Figure 1). Intakes for a young infant and adolescent based on a typical diet were significantly higher at 42.1 and 8.2 μg/kg-day, respectively. USDA estimates were lower for infants at 37.4 μg/kg-day but higher for adolescent girls, 10.8 μg/kg-day, and boys, 11.7 μg/kg-day. High consumption of meat and dairy also resulted in the highest DEHP exposures in adolescents (21.6 μg/kg-day) and infants (90.6 μg/kg-day) in comparison to the other dietary patterns. A diet high in fresh vegetable and fruit intake resulted in slightly lower exposures than actual diets for infants but higher exposures for adolescents and females of reproductive age (infants: 40.1; adolescents: 12.1; females of reproductive age: 6.0 μg/kg-day) (Figure 1).

**Table 3:** **Per capita total DEHP dietary intake for eight major food groups in average diets of US infants, adolescents and females of reproductive age**^**a**^

**Food group**	**Conc.**^**b **^**(μg/kg)**	**Young infants (1–2 years)**		**Adolescents (13–19 years)**		**Females of reproductive age (13–49)**	
		**Food consumption**^**c **^**(g/kg-day)**	**Daily intake (%) (μg/kg-day)**	**Food consumption**^**c **^**(g/kg-day)**	**Daily intake (%) (μg/kg-day)**	**Food consumption**^**c **^**(g/kg-day)**	**Daily intake (%) (μg/kg-day)**
Total dairy	712.4	43.2	30.8 (73.1)	5.5	3.9 (47.9)	3.8	2.7 (47.2)
Total meat	209.6	4	0.8 (2.0)	2	0.4 (5.1)	1.6	0.3 (5.8)
Total egg	21.1	1.40^d, e^	0.03 (0.1)	0.25^d, f^	0.01 (0.1)	0.23^d, g^	0.01 (0.1)
Total fish	180.4	0.26	0.05 (0.1)	0.13^h^	0.02 (0.3)	0.19	0.03 (0.6)
Total grain	187.4	6.4	1.2 (2.8)	2.4	0.5 (5.5)	1.9	0.04 (6.2)
Total vegetable	131.9	6.7	0.9 (2.1)	2.3	0.3 (3.7)	2.5	0.3 (5.8)
Total fruit	115.6	7.8	0.9 (2.1)	0.9	0.1 (1.3)	1	0.1 (2.0)
Total fat	1851.7	4	7.4 (17.6)	1.6^h, i^	3.0 (36.2)	1^i, j^	1.9 (32.3)
Total dietary intake	3409.7	73.76	42.1 (100)	15.08	8.2 (100)	12.22	5.7 (100)

^a^(Concentration in Food/1000) *Daily Food Consumption = Daily Intake.

^b^Weighted average of all available mean concentrations in foods corresponding to one of the eight food categories. Calculated by taking the sum of each average concentration multiplied by individual number of samples and diving by total number of samples: $\left(\left(\mathrm{avg}.\mathrm{conc}.\mathrm{dairy}1*n\right)+\left(\mathrm{avg}.\mathrm{conc}.\mathrm{dairy}2*n\right)\ldots \right)/\sum n$.

^c^Source NHANES 2003–2006.

^d^Source USDA CSFII 1994–1996, 1998.

^e^Calculated for a 11.4 kg infant.

^f^Calculated for adolescent under 19 years old and 56.8 kg.

^g^Calculated for female 20 and over and 70 kg.

^h^11 to <21 years.

^i^Source NHANES 2007.

^j^Females 21 to <41 years.

## Discussion

The review of the literature revealed that poultry, some dairy products (cream) and fats are routinely contaminated with high concentrations of DEHP than other foods. Milk, yogurt, eggs, fruits, vegetables, pasta, noodles, rice, beverages and water were found to contain low concentrations of phthalates as a whole.

Given the chemistry of high molecular weight phthalates like DEHP, higher concentrations in lipid rich foods were expected. There was significant variability in concentration observed between dairy products based on typical fat content. Among the dairy products tested, cream and cheese were more heavily contaminated across studies in comparison to yogurt. Poultry consistently had higher phthalate content than other meats, however it is unclear what factors impacted these results since details as to the fat content of products was not always reported. Noteworthy, phthalates in non-fatty foods including bread and cereal products were observed in variable concentrations. This is of importance since two recent studies conducted in Belgium and Germany reported bread as a significant source of DEHP and highest contributor to total exposures in the general adolescent and adult population at 31.4% and 14.06%, respectively [54,55]. Sources of contamination may be present in the processing of grains, though this is unclear. As a whole, food monitoring data also suggests that the consumption of fruit and vegetables is associated with limited phthalate exposures. However, processed fruit and vegetable products found in jars appear to contribute to greater exposures given the high concentrations reported.

As expected, the epidemiology literature reported that dairy products, meats and discretionary fat intake, in fact, were associated with increases in DEHP urinary metabolite levels in adolescent and adult populations. Further, consumption of these products were found to be associated with MnBP levels in one epidemiology study and elimination of some of these products from the diet (dairy and meat) led to a decrease in MnBP and DEHP metabolites in the Temple Stay intervention [50,51]. It is also possible that the consumption of dairy products in Sathyanarayana’s intervention study may have contributed to increased MnBP levels; however, this is not entirely clear since foods were not analyzed for this particular parent compound. It is important to note that although results from Ji and others suggest that discontinuing meat and dairy from the diet may be largely responsible for decreases in metabolite levels, there may have been other factors in the environment that impacted results since details of the diet as well as daily practices in the Temple Stay program were not available [51]. It is possible that decreases in low molecular weight phthalates, given their primary source, could be attributed to reduced use of personal care products rather than changes in the diet [51,56].

Results between food monitoring and epidemiological data were not completely consistent. Two epidemiology studies reported an association between fish consumption and MiBP; however the food monitoring data did not support this result as all DiBP levels in seafood were found to be low across studies. Additionally, increased levels of MMP (a metabolite of DMP) were associated with consumption of fruit while the food monitoring data did not show DMP at significant levels for this food group [26]. Finally, Colacino et al. as well as Trasande et al. reported positive associations between vegetables and MEP. However, the food monitoring data does not support this finding and two other epidemiology studies (Dickson-Spillman et al. and Ji et al.) suggest that diets with high consumption of fruits and vegetables may be associated with decreases in DEP exposure. It is important to note, that DEP (as well as DiBP and DMP) have other non-dietary sources which were not accounted for in the epidemiology studies reviewed. Identifying the relative contribution of different phthalate sources is a gap in the current literature and will require more comprehensive assessment of individual behaviors related to diet, personal care product use, and occupation.

Given the paucity in US data and an increasing global food market, we included European and Asian studies in our average calculation of phthalate concentrations in food. In comparison to current guidelines, dietary DEHP intake estimates based on typical diets for women of reproductive age and adolescents were below the EPA’s reference dose of 20 μg/kg-day for risk of increased liver weight and the EFSA’s total daily intake of 50 μg/kg-day for developmental risk of testicular toxicity [57,58]. However, the exposure estimate for a typical diet in infants exceeded the EPA’s reference dose (42.1 μg/kg-day) while a diet high in meat and dairy was over this threshold by approximately four times. For adolescents, a diet high in meat and dairy also exceeded the EPA’s reference dose. All diets for all groups exceeded the allowable daily intakes (ADI) derived by the US Consumer Product Safety Commission (CPSC) for the risk of aspermatogenesis (5.8 μg/kg-day) while diets high in meat and dairy consumption exceeded the ADI for reproductive malformations in females (11.5 μg/kg-day) [59]. Surprisingly, diets high in fresh fruits and vegetables resulted in greater DEHP exposure than actual consumption patterns for adolescents and women of reproductive age. This higher exposure is most likely due to increased dairy consumption within this dietary pattern from 5.5 to 9 g/kg-day in adolescents and 1.6 to 2 g/kg-day in females of reproductive age. Given that these estimates are solely based on dietary intake, we expect total exposure to be greater due to other sources of DEHP in the environment that were not accounted for in this calculation.

### Future research

The review of the available literature on dietary phthalate exposure allowed us to identify gaps in the research that may be addressed in future studies. Most surveys reported on raw foods taken directly out of packaging without preparation but this does not reflect typical diets that normally have an abundance of cooked/prepared foods. Fierens and colleagues investigated the effect of cooking (boiling, steaming, frying or grilling) at home on the levels of phthalates in various food types (starchy products, vegetables, meat and fish [44]. In general, phthalate concentrations in foods declined after cooking, except in vegetables, where almost no effect was seen. DEHP was present in all raw foodstuffs, although the percentage decreased to 65.4% after cooking [44]. Another study in Italy showed that cooked nursery and primary school lunches had higher DEHP and DBP concentrations after being packaged in polyethylene-coated aluminum dishes and kept warm on electrically powered isotherm serving carts in comparison to before, suggesting migration from packaging [60]. Therefore, the impact of cooking as well as heating in phthalate-containing containers needs to be considered in future calculated exposure assessments in order to capture true phthalate content in food that is directly consumed. One study was identified that measured daily intake in a Belgian population taking food preparation into account; investigators showed declines in DiBP and DEHP exposure due to cooking [54].

It is also necessary to differentiate between products within some food categories when calculating daily intakes (i.e. dairy, meats, grains) in order to understand what product has the highest contribution of phthalate to overall exposure. This is especially important when providing recommendations for the types of high phthalate exposure foods to avoid. Two intervention studies that focused on the elimination of packaging as a method to reduce dietary phthalate exposure had varying results. Products that were suspected of being responsible for the increases in metabolite levels in the Sathyanarayana study were dairy (butter, heavy cream, cheese, and milk) and spices (coriander, cayenne and cinnamon). Butter and heavy cream were reported to be used in some of the largest quantities in foods prepared by the caterer. In comparison, the Rudel study provided dairy products that included Swiss cheese, milk and yogurt while there were no reports of the use of specific spices. It is therefore possible that the type of food rather than the packaging may have impacted exposures in these studies. It would be of benefit to compare phthalate concentrations of specific foods that are found both fresh and as packaged/processed products in order to understand the extent at which packaging impacts phthalate content versus pre-market processes. Of the limited information available in this review, we found higher concentrations in frozen fish but lower concentrations in frozen beef in comparison to their fresh or non-frozen counterparts. The limited epidemiology studies and some of the food monitoring surveys did not include DiNP or its metabolite, MCOP nor DiDP or its metabolite, MCNP. Of the food monitoring data that was available, DiNP was found in high individual concentrations in some foods including chicken thighs (1819.6 μg/kg), craster kipper fillets (11,576 μg/kg) and dairy butter (1499.6 μg/kg) within a UK study [38]. However, within this same study, limits of detection or quantification were extremely high which the authors attributed to the use of liquid chromatography-mass spectrometry (LC-MS) methods rather than gas chromatography–mass spectrometry (GC-MS). This may lead to misleading results. Further, there is concern over DiNP since as of December 20, 2013 the Office of Environmental Health Hazard Assessment (OEHHA) of California added the phthalate species to the list of chemicals known to the state to cause cancer. Given the toxicity and significant increase in use as replacements for DEHP within the past decade as well as the lack of reliable data, future research should assess DiNP as well as DiDP dietary exposure utilizing appropriate analytical techniques. We observed significant differences in calculated daily DEHP exposures based on varying consumption of foods. Therefore, epidemiology studies that investigate the association between particular types of dietary patterns and phthalate metabolite urinary biomarkers are needed to corroborate results. Finally, given multiple sources of exposure, a comprehensive assessment taking all routes into account is needed for an American population.

## Conclusion

DEHP in some meats, fats and dairy products were found at high concentrations (≥300 μg/kg) in food monitoring surveys and significantly contributed to exposure in epidemiological studies. Similarly, assessment of daily dietary DEHP intake resulted in dairy as the highest contributor to exposure. Exposure estimates based on actual diets for infants exceeded the Environmental Protection Agency’s reference level while estimates based on high meat and dairy consumption resulted in exposure above this limit for adolescents. Some of the ADI’s developed by the CPSC for reproductive outcomes were also exceeded. We provide guidance on future research in this area to further understand food as an important phthalate source and to help identify methods to reduce dietary phthalate exposures.

## Abbreviations

ADI: Allowable daily intake; AGD: Anogenital distance; BBzP: Benzylbutyl phthalate; BMI: Body mass index; CFSII: Continuing survey of food intake by individuals; CI: Confidence interval; CPSC: Consumer product safety commission; DEP: Diethyl phthalate; DEHP: Di-2-ethylhexyl phthalate; DiBP: Di-isobutyl phthalate; DiDP: Di-isodecyl phthalate; di-isononyl phthalate; DnBP: Di-n-butyl phthalate; DnOP: Di-n-octyl phthalate; DMP: Dimethyl phthalate; EFSA: European food safety authority; EPA: Environmental protection agency; FDA: Food and drug administration; FID: Flame ion detector; GC-MS: Gas chromatography–mass spectrometry; LC-MS: Liquid-chromatography-mass spectrometry; LOD: Limit of detection; LOQ: Limit of quantification; MBzP: Mono-benzyl phthalate; MCNP: Mono-carboxynonyl phthalate; MCOP: Mono-carboxyoctyl phthalate; MCPP: Mono-(3-carboxypropyl) phthalate; MEP: Mono-ethyl phthalate; MEHP: Mono-2-ethylhexyl phthalate; MEHHP: Mono-(2-ethyl-5-hydroxyhexyl) phthalate; MECPP: Mono-(2-ethyl-5-carboxypentyl) phthalate; MEOHP: Mono-(2-ethyl-5-oxohexyl) phthalate; MiBP: Mono-isobutyl phthalate; MnBP: Mono-n-butyl phthalate; MnMP: Mono-n-methyl phthalate; NHANES: National health and nutrition examination survey; PID: Photo ion detector; PVC: Polyvinyl chloride; OEHHA: Office of environmental health hazard assessment; SML: Specific migration limit; UK: United Kingdom; US: United States; USDA: United States department of agriculture.

## Competing interests

The authors declare that they have no competing interests.

## Author’s contributions

SES and SS reviewed and summarized the literature. RD consulted on appropriate analytical methods of reviewed studies. SES and SS calculated daily DEHP intakes. SES drafted the manuscript and SS, JB, and LT contributed to its content. All authors read and approved the final manuscript.

## Supplementary Material

Additional files 1: Table S1 Average concentrations of phthalates (μg/kg) in various foods and beverages. Click here for file
